# Transcriptional Regulation of Aluminum-Tolerance Genes in Higher Plants: Clarifying the Underlying Molecular Mechanisms

**DOI:** 10.3389/fpls.2017.01358

**Published:** 2017-08-08

**Authors:** Abhijit A. Daspute, Ayan Sadhukhan, Mutsutomo Tokizawa, Yuriko Kobayashi, Sanjib K. Panda, Hiroyuki Koyama

**Affiliations:** ^1^Faculty of Applied Biological Sciences, Gifu University Gifu, Japan; ^2^Faculty of Life Science and Bioinformatics, Assam University Silchar, India

**Keywords:** Al, ALMT1, phytohormone, ROS, STOP1

## Abstract

Aluminum (Al) rhizotoxicity is one of the major environmental stresses that decrease global food production. Clarifying the molecular mechanisms underlying Al tolerance may contribute to the breeding of Al-tolerant crops. Recent studies identified various Al-tolerance genes. The expression of these genes is inducible by Al. Studies of the major *Arabidopsis thaliana* Al-tolerance gene, *ARABIDOPSIS THALIANA ALUMINUM-ACTIVATED MALATE TRANSPORTER 1 (AtALMT1)*, which encodes an Al-activated malate transporter, revealed that the Al-inducible expression is regulated by a *SENSITIVE TO PROTON RHIXOTOXICITY 1* (*STOP1*) zinc-finger transcription factor. This system, which involves STOP1 and organic acid transporters, is conserved in diverse plant species. The expression of *AtALMT1* is also upregulated by several phytohormones and hydrogen peroxide, suggesting there is crosstalk among the signals involved in the transcriptional regulation of *AtALMT1*. Additionally, phytohormones and reactive oxygen species (ROS) activate various transcriptional responses, including the expression of genes related to increased Al tolerance or the suppression of root growth under Al stress conditions. For example, Al suppressed root growth due to abnormal accumulation of auxin and cytokinin. It activates transcription of *TRYPTOPHAN AMINOTRANSFERASE OF ARABIDOPSIS 1* and other phytohormone responsive genes in distal transition zone, which causes suppression of root elongation. On the other hand, overexpression of Al inducible genes for ROS-detoxifying enzymes such as *GLUTATHIONE–S-TRANSFERASE*, *PEROXIDASE*, *SUPEROXIDE DISMUTASE* enhances Al resistance in several plant species. We herein summarize the complex transcriptional regulation of an Al-inducible genes affected by STOP1, phytohormones, and ROS.

## Introduction

The insoluble oxidized form of aluminum (Al) in soil clay becomes soluble in acidic soil solutions at pH < 5.5 (Kinraide and Parker, 1989). Among the various Al ion species, Al^3+^ is the most toxic form. This ion can exist in naturally acidic soils, and is toxic at sub-micromolar concentrations (Kinraide et al., 1985; Kinraide, 2003). Crop growth is severely suppressed in Al-solubilizing soils. This is primarily because of the root growth inhibition due to Al rhizotoxicity (i.e., cytotoxic and genotoxic effects of Al^3+^), which restricts root tip cell elongation and division (Nezames et al., 2012). Improving crop tolerance to Al rhizotoxicity is one of the most important targets for increasing crop production in regions with acidic soil, which are dominant in the sub-tropical and tropical regions of many highly populated developing countries. Recent studies revealed that the transcriptional regulation of stress-tolerance genes is important for Al tolerance (Kochian et al., 2015).

Differences in the expression levels of Al-tolerance genes influence the extent of Al tolerance in several important crops such as wheat (Sasaki et al., 2004), rice (Yamaji et al., 2009), and sorghum (Magalhaes et al., 2007). Additionally, an analysis of expression level polymorphisms among *Arabidopsis thaliana* accessions identified several novel genes that regulate Al tolerance (Kusunoki et al., 2017). In contrast, the identification and characterization of transcription factors and regulatory proteins (e.g., protein kinases) revealed that the transcription of Al-tolerance genes is likely regulated by a very complex mechanism. This mechanism involves repressors and activators, co-regulation with other Al-tolerance genes (e.g., Delhaize et al., 2012; Tokizawa et al., 2015), and crosstalk with mechanisms controlling other stress responses. A more comprehensive characterization of these complex regulatory mechanisms may be useful for accelerating the breeding of Al-tolerant crops.

The different types of Al-tolerance mechanisms in various crops involve the exclusion of Al, an internal tolerance mechanism, and recovery from Al-induced damages (Taylor, 1987, 1988, 1991; Kochian, 1995). The excretion of Al-detoxifying ligands, such as organic acids (OAs) and *P*_i_, to the apoplast or rhizosphere is the most common feature of Al-exclusion mechanisms in several crop plants. The OA types differ among plant species (Ma et al., 1998; Zheng et al., 1998; Wenzl et al., 2001; Kobayashi et al., 2005). The internal tolerance mechanisms involve the sequestration of Al into vacuoles and the detoxification of Al by chelation. Meanwhile, recovery from Al-induced damages is mediated by the detoxification of the reactive oxygen species (ROS) produced following exposures to excessive amounts of Al. By combining these mechanisms with the transcriptional regulation of Al-tolerance genes, plants can protect the most sensitive part of the root apex from Al rhizotoxicity. The expression of genes encoding OA transporters is inducible by Al. These genes include *ALUMINUM-ACTIVATED MALATE TRANSPORTER 1* (*ALMT1*) and members of the multidrug and toxic compound extrusion (MATE) citrate transporter gene family in *A. thaliana* (Sawaki et al., 2009) and tobacco (*Nicotiana tabacum*; Ohyama et al., 2013). A previous study of *A. thaliana* detected a typically complex regulation of the Al-inducible expression of *AtALMT1*, which encodes a protein that mediates protein phosphorylation/dephosphorylation processes (Kobayashi et al., 2007). Other studies concluded that *AtALMT1* expression is regulated by transcription factors (Ding et al., 2013; Tokizawa et al., 2015) and phytohormone-signaling networks (e.g., jasmonate and ethylene; Kobayashi et al., 2013). The expression of *AtALMT1* is tightly regulated by the STOP1 (sensitive to proton rhizotoxicity 1; Iuchi et al., 2007) zinc-finger transcription factor, and is co-regulated with several other Al- and proton-tolerance genes (Sawaki et al., 2009). This complex regulation may account for the pleiotropic roles of ALMT1 (Koyama et al., 2015).

**FIGURE 1:**
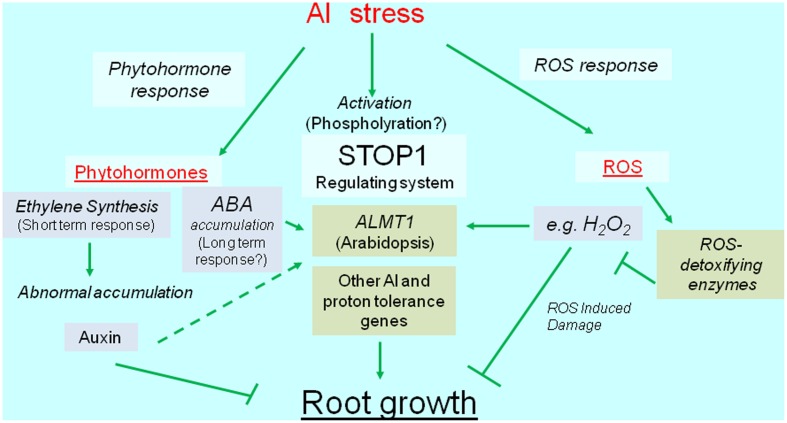
Diagrammatic representation of transcriptional regulation of Al tolerance genes. Al stress activates transcription of Al tolerance genes (green box), which are regulated the transcription via phytohormone, SENSITIVE TO PROTON RHIZOTOXICITY1 (STOP1), and reactive oxygen species (ROS) mediated pathways. The major Al tolerance gene, such as *ALUMINUM ACTIVATED MALATE TRANSPORTER1* (*ALMT1*) -type malate transporter, is regulated the transcription by STOP1-like protein, but it’s likely activated by phytohormone(s) and ROS. ROS induces various genes including ROS detoxifying enzymes.

Reactive oxygen species can cause irreversible damage to growing tissues, in part because of Al-induced metabolic changes. This damage induces the transcription of genes encoding ROS-detoxifying enzymes (e.g., glutathione *S*-transferase, peroxidase, alternative oxidase, and malate dehydrogenase). The overexpression of these genes usually confers Al tolerance, which suggests that plants activate Al-tolerance mechanisms to recover from Al-induced ROS damages. Additionally, recent developments in next-generation sequencing technologies have resulted in the identification of several novel Al-inducible genes that influence the level of Al tolerance (Kusunoki et al., 2017). Overall, we speculate that transcriptional regulation is critical for mechanisms mediating the Al tolerance of crops (**Figure 1**). We herein summarize our current understanding of the transcriptional regulation of Al-tolerance genes and its relevance to the breeding of Al-tolerant varieties.

## Transcriptional Regulation of Al-Tolerance Genes by the Stop1 Protein

The *SENSITIVE TO PROTON RHIZOTOXICITY (STOP1)* gene was discovered based on the positional cloning of an *A. thaliana* mutant with short roots in an acidic medium (Iuchi et al., 2007). The mutant was also hypersensitive to Al because of the suppressed expression of *AtALMT1* (Iuchi et al., 2007). In *A. thaliana*, the Al-inducible expression of *AtALMT1* is critical for Al tolerance (Hoekenga et al., 2006), while the expression of *AtALMT1* is completely suppressed in the *stop1* mutant (Iuchi et al., 2007). STOP1 contains four zinc-finger domains, suggesting it is a critical transcription factor regulating the expression of *ALMT1* and proton-tolerance genes. Additionally, *AtALMT1* is co-regulated with genes for proton tolerance under the control of STOP1, which is a protein that is essential for *AtALMT1* transcription. Functional orthologs of AtSTOP1 are key regulators of Al-tolerance genes in various plant species. In this section, we describe our current understanding of the STOP1-like protein, including its effects on Al-inducible expression of Al-tolerance genes.

### STOP1-Regulated Genes in *Arabidopsis thaliana*

A systems biology study uncovered the co-regulation of multiple Al- and proton-tolerance genes by AtSTOP1 in *A. thaliana*, including *ALUMINUM SENSITIVE 3* (*ALS3*; possibly encodes UDP glucose transporter, homolog of rice *STAR2*) (i.e., Al tolerance) and various genes affecting ion homeostasis [e.g., *CBL-INTERACTING PROTEIN KINASE 23* (*CIPK23*), which phosphorylates *ARABIDOPSIS POTASSIUM TRANSPORTER1*; Xu et al., 2006], pH-regulated metabolic activities (e.g., GABA-shunt and biochemical pH stat pathways), and cell wall stabilization (i.e., proton tolerance) (Sawaki et al., 2009). Additionally, Al-inducible expression of *MULTIDRUG AND TOXIC COMPOUND EXTRUSION (AtMATE;* encoding a citrate transporter) is regulated by STOP1 (Liu et al., 2009). These results indicate that STOP1 is a key regulatory transcription factor for Al and proton tolerance.

**FIGURE 2:**
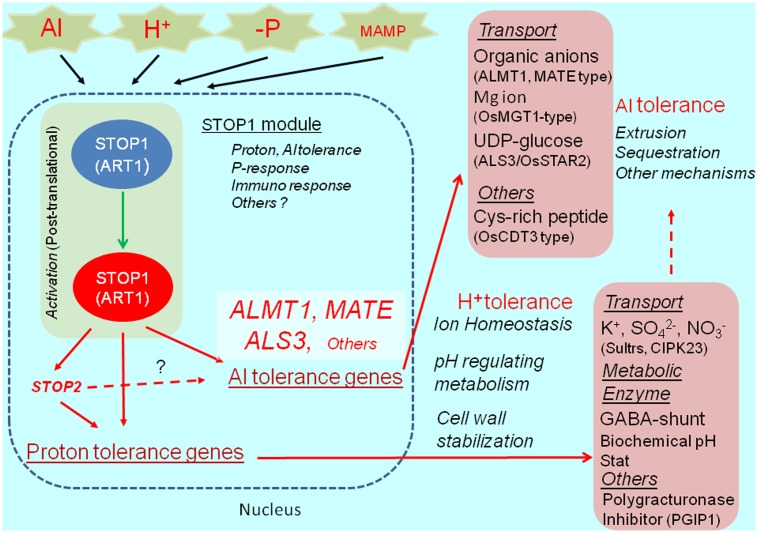
STOP1 module and *ALMT1* regulation. The SENSITIVE TO PROTON RHIZOTOXICITY1 (STOP1) module, which is consisted of STOP1-like protein (in Arabidopsis, STOP1; in rice ALUMINUM RESISTANCE TRANSCRIPTION FACTOR 1), OA transporters *ALUMINUM ACTIVATED MALATE TRANSPORTER1* (*ALMT1*), malate transporter; *MULTIDRUG AND TOXIC COMPOUNDS EXTRUSION* (*MATE*); citrate transporter, and *ALUMINUM SENSITIVE 3* (*ALS3*), are conserved in wide range of plant species. The module includes various Al tolerance genes and possibly H^+^ tolerance genes (pink box), which play critical role in Al detoxification, ion homeostasis (transport and metabolisms), cell wall stabilization and other roles for tolerance. In Arabidopsis, STOP1 regulates *STOP2* expression, while STOP2 only regulates expression of H^+^ tolerance genes. STOP1-regulating gene, such as ALMT1, is activated by various environmental stimuli (Al, H^+^ -P and MAMP, Microbe Associated Molecular Pattern).

The Al- and proton-tolerance genes are differentially regulated by STOP1. In *A. thaliana*, STOP2 is a unique ortholog of STOP1, with a shorter C-terminus. It rescues the proton tolerance in the *stop1* mutant by activating the expression of several proton-tolerance genes (e.g., *CIPK23* encoding regulator of K^+^ and NO^3-^ transporters, *polygalacturonase inhibitor protein 1*, stabilizing pectin at low pH, and others; Kobayashi et al., 2014). Although *STOP2* expression is regulated by STOP1, it does not recover Al tolerance in the *stop1* mutant because of the very limited recovery of *AtALMT1* and *ALS3* expression. This suggests that STOP2 only enhances the proton-tolerance mechanism controlled by STOP1. Additionally, an *in planta* complementation of STOP1-like proteins usually fails to recover Al tolerance in the *A. thaliana stop1* mutant, while it confers proton tolerance (e.g., Ohyama et al., 2013). These observations suggest that the transcriptional activation of major Al-tolerance genes by STOP1 (e.g., *AtALMT1* and *ALS3*) requires additional mechanisms (e.g., co-activators or post-translational mechanisms), which are sensitive to the STOP1 protein structure (**Figure 2**).

### Conservation of the STOP1-Regulated System in Various Plant Species

The *ALUMINUM-RESISTANCE TRANSCRIPTION FACTOR1* (*OsART1*) gene is a rice ortholog of *AtSTOP1* that was identified during the positional cloning of an Al-sensitive mutant (Yamaji et al., 2009). The *art1* mutant exhibits repressed expression of *STAR2* [(rice homolog of *AtALS3* (Larsen et al., 2005; Yamaji et al., 2009)] and *Oryza sativa FERRIC REDUCTASE DEFECTIVE 4* (*OsFRDL4)* (rice homolog of *AtMATE*; Yamaji et al., 2009). Additionally, a magnesium transporter (Chen et al., 2012) and a plasma-membrane–localized cysteine-rich peptide CDT3 (Xia et al., 2013; see also in ROS section) are critical for Al resistance in rice, and the expression of the corresponding genes is regulated by the ART1 transcription factor. These observations strongly suggest that a STOP1-like protein regulates the expression of multiple Al-tolerance genes in various plant species. In fact, functional orthologs of STOP1 (hereafter called STOP1-like proteins) have been identified in a wide range of plant species, including dicotyledons and monocotyledons, tree and grass species, *Brassicaceae* species, legumes, and mosses (Ohyama et al., 2013; Sawaki et al., 2014; Fan et al., 2015; Wang et al., 2017). A *Physcomitrella patens* subsp. *patens* knock-out of *STOP1* is reportedly more sensitive to Al than the wild-type (WT) plants. These findings indicate that STOP1-like protein(s), and regulating core Al tolerance gens (STOP1-module), are conserved among land plant species.

The suppression of *NtSTOP1* expression (*NtSTOP1*-KD) based on RNA interference represses the Al tolerance of tobacco plants (*N. tabacum*) because the Al-inducible expression of *NtMATE1* is inhibited (Ohyama et al., 2013). The *NtMATE1* expression level in *NtSTOP1*-KD plants is more than 100-fold lower than in WT plants. Additionally, Al accumulates in the root tips of the *NtSTOP1*-KD plants (see Figure 4A in Ohyama et al., 2013). This complete suppression of *NtMATE* expression is very similar to the lack of *AtAMLT1* expression in the *stop1 A. thaliana* mutant. In contrast, the Al-inducible expression of *NtALS3* is regulated by NtSTOP1 (Ohyama et al., 2013). Thus, the STOP1-like protein tightly regulates the genes encoding OA transporters and ALS3 orthologs, with diverse transporters likely being regulated in different plant species. The Al-inducible transcription of genes encoding OA-transporters involves a STOP1-binding process. A yeast one-hybrid assay revealed that VuSTOP1 binds to the promoter of *VuMATE1* to regulate expression (Fan et al., 2015). Gel-shift assays involving OsART1 confirmed that GGN(T/g/a/C)V(C/A/g)S(C/G) (simply, GGNVS-consensus) is the canonical OsART1-binding sequence in the promoters of 29 of the 31 genes regulated by ART1 (e.g., *OsFRDL4*) (Tsutsui et al., 2011). In contrast, the *AtALMT1* promoter carries a STOP1-binding site that is longer (about 15 bp) than the corresponding rice sequence. This binding site is suitable for the four zinc-finger domains of the STOP1-like protein (Tokizawa et al., 2015). Several studies concluded that the number of STOP1-binding sites in the promoter influences the OA transporter gene expression-level differences among cultivars (Chen et al., 2013). A *Holcus lanatus* accession adapted to an acid plot was observed to carry several GGN(T/g/a/C)V(C/A/g)S(C/G) sequences in the *ALMT1* promoter (Chen et al., 2013), while wheat near-isogenic line ET8, which exhibits upregulated *TaALMT1* expression, carries three sets of the STOP1-binding site identified in the Al-sensitive near-isogenic line ES8 (Tokizawa et al., 2015) (**Figure 2**).

### Complexity of the STOP1-Regulated Expression of OA Transporter Genes, and the Associated Pleiotropic Functions

Recent studies have confirmed that the mechanism underlying the STOP1 regulation of the expression of OA transporter genes is complex because several other transcription factors are involved. The Al-inducible *AtALMT1* expression is associated with the upregulated expression of calmodulin-binding transcription activator 2 (Tokizawa et al., 2015) and the downregulated expression of *AtWRKY46*, which encodes a repressor of *AtALMT1* expression (Ding et al., 2013). abscisic acid (ABA) induce the *AtALMT1* expression (see the below; Phytohormone section), while the promoter deletion analysis identified that differential regulation of transcription by ABA and Al in short term (6 h, Kobayashi et al., 2013). On the other hand, *AtWRKY46* reported as repressor of *AtALMT1* (Ding et al., 2013) and ABA repress the expression of *AtWRKY46* (Ding et al., 2015). Furthermore, analyses of the *AtALMT1* promoter (i.e., bioinformatics based promoter-GUS reporter assay) identified multiple *cis*-elements responsible for the short- and long-term Al-inducible expression (Tokizawa et al., 2015). The *cbl1* mutant exposed to Al stress conditions reportedly exhibits inhibited root growth, decreased malate secretion, and increased accumulation of Al in the root tips (Ligaba-Osena et al., 2017). This suggests that the *ARABIDOPSIS THALIANA CALCINEURIN B-LIKE PROTEIN 1* (*AtCBL1*) gene product helps activate STOP1 in *A. thaliana*. Because CBL1 activates several regulatory protein kinases, it may also be involved in the protein phosphorylation that activates *AtALMT1* expression (Kobayashi et al., 2007). However, as described above, the *in planta* complementation of STOP1-like proteins in the *A. thaliana stop1* mutant often fail to induce *AtALMT1* transcription, while activating the expression of proton-tolerance genes. This also applies to the functional ortholog, NtSTOP1, which can activate *NtMATE* expression in tobacco (Ohyama et al., 2013). The reasons for these observations may be related to the differential structures of the N- and C-termini, which are specifically targeted for post-translational modifications or for interactions with a co-activator of *AtALMT1* expression.

Although the relevant regulatory mechanisms have not been fully characterized, STOP1 and ALMT1 have pleiotropic roles related to adaptations to other stressors. Rudrappa et al. (2008) reported that infections to the aerial parts of *A. thaliana* plants by pathogenic bacteria upregulate *AtALMT1* expression and malate excretion in the roots. The excreted malate recruits beneficial rhizobacteria that stimulate the *A. thaliana* immune system. The molecular mechanism underlying the long-distance signaling from the shoots to the roots has not been elucidated. However, *AtALMT1* expression is induced in roots treated with the FLG22 peptide (i.e., conserved peptide in the bacterial flagella), which likely involves the FLG22 receptor FLAGELLIN-SENSITIVE 2, a type of MAMP (microbe associated molecular pattern) (Kobayashi et al., 2013). Additionally, a recent study determined that STOP1 and ALMT1 trigger a malate-exudate–dependent Fe relocation in the root apical meristem, which is essential for the reprogramming of root growth under low-Pi conditions (Balzergue et al., 2017; Macias et al., 2017). The fact that *AtALMT1* expression is dependent on STOP1 binding to the promoter suggests that STOP1 is a HUB molecule of these multiple responses. Al induces malate excretion *via STOP1/ALMT1* activities, with pleiotropic consequences for stress tolerance. Further research is required to uncover the molecular mechanisms related to the activation of STOP1.

## Phytohormones Involved in Al-Stress Responses and their Role in the Transcription of Al- and Proton-Tolerance Genes

**FIGURE 3:**
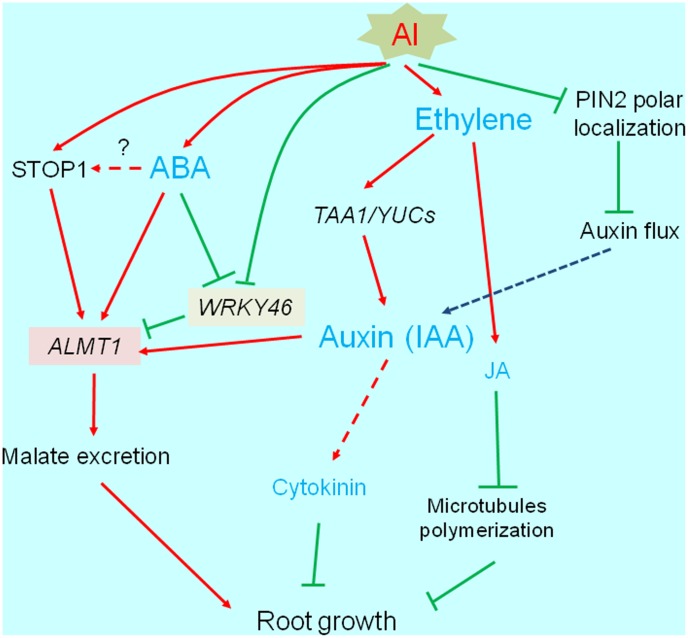
Transcriptional regulation of phytohormone responsive root growth under Al stress. Al induces root growth inhibition, which is associated with increased ethylene synthesis followed by accumulation of auxin (IAA) and jasmonic acid (JA). These events occur within short-term range (3 h), which is associated with *PIN-FORMED* (PIN2). Finally, IAA and JA activated pathways are likely blocking root growth. IAA and abscisic acid activate *ALUMINUM ACTIVATED MALATE TRANSPORTER1* (*ALMT1*) expression, which may increase Al tolerance.

Most phytohormones are important for root development and elongation (see reviews; Benkova and Hejatko, 2009; Jung and McCouch, 2013). Additionally, phytohormones help mediate biotic and abiotic stress responses (e.g., by activating the transcription of various genes). Many studies have revealed that several phytohormones affect the Al-induced inhibition of root growth. The abnormal accumulation of phytohormones disrupts normal root growth. Additionally, the accumulated phytohormones modify the transcriptome, and affect the activation of some Al-tolerance genes. In this section, we describe the relationships between phytohormones and plant responses to Al stress by categorizing the phytohormones according to whether they negatively or positively affect root growth under Al stress conditions (**Figure 3**).

### Negative Effects of Phytohormones on Root Growth under Al Stress Conditions and the Consequences for Transcriptional Regulation

Auxin is a key regulatory phytohormone for root development and elongation. The auxin gradient in the root apex is formed by a series of auxin polar transporters such as the PIN-FORMED (PIN) family proteins (Blilou et al., 2005; Leyser, 2005; Baluska et al., 2010; Takatsuka and Umeda, 2014). The root apex auxin gradient along with the high and low auxin concentrations in the meristem and elongation zone, respectively, are essential for continuous root growth. Normal root growth is disrupted by Al stress conditions, especially in the distal transition zone (DST) (i.e., 1–3 mm behind the root tip, corresponding to the transition from cell division to cell elongation; Kollmeier et al., 2000). Moreover, Al interferes with the plasma membrane localization of PIN2, which regulates the auxin flux from the root tip to the elongation zone (Shen et al., 2008). These results imply that the disruption of PIN-mediated auxin flow is one of the causes of inhibited root growth due to Al toxicity. This possibility is supported in *A. thaliana* and rice by the observed changes in the Al-sensitivity of mutants as well as the phenotypes of transgenic plants overexpressing the genes encoding certain PIN proteins (Sun et al., 2010; Wu et al., 2014, 2015). Yang et al. (2014) characterized the mechanisms regulating the indole-3-acetic acid (IAA)-mediated inhibition of root growth under Al stress conditions. They determined that the Al-induced accumulation of IAA in the DST of *A. thaliana* plants is caused by TAA1-mediated auxin biosynthesis, which is concomitant with activation of transcription of other genes for IAA synthesizing proteins (e.g., YUCCA; Liu et al., 2016). The inhibition of root elongation by accumulating IAA occurs simultaneously with the increased production of ethylene in response to Al stress. This increase in ethylene contents suppresses the expression of cell-wall modification genes mediated by auxin-response factors 10 and 16 (Yang et al., 2014). Ethylene is a key regulator of auxin biosynthesis and basipetal auxin transport in the root apex (Ruzicka et al., 2007; Stepanova et al., 2007; Swarup et al., 2007; Muday et al., 2012). Thus, it can inhibit root growth in Al-stressed plants. In fact, Al induces rapid and considerable increases in ethylene levels in *A. thaliana* (Sun et al., 2010), *Lotus japonicus* (Sun et al., 2007), and *Phaseolus vulgaris* (Massot et al., 2002). This increase is due to the upregulated expression of genes encoding ethylene biosynthesis enzymes, including ACC-synthase and ACC-oxidase (Sun et al., 2010). An *in planta* GFP reporter assay involving the auxin-responsive promoter (DR5) confirmed that Al-inducible IAA accumulation can be suppressed by the ethylene synthesis inhibitor aminoethoxyvinylglycine (Yang et al., 2014). This inhibition is induced after a 1.5-h exposure to Al, which follows a very quick Al inhibition (initiated at 5 min), possibly via the inhibition of cell-wall loosening resulting from the binding of Al (Kopittke et al., 2015). Yang et al. (2017a,b) also demonstrated that Al-induced upregulation of ethylene synthesis suppresses root growth by mediating the jasmonic acid (JA)-responsive and cytokinin-responsive pathways. The accumulation of JA-isoleucine (i.e., active form of JA) in the root tips is induced by Al, but is suppressed by aminoethoxyvinylglycine. The accumulated JA-isoleucine downregulates the expression of microtubule-associated genes, resulting in inhibited root growth (Yang et al., 2014).

Indole-3-acetic acid can activate the transcription of Al-tolerance genes. For example, the application of exogenous IAA induces considerable increases in *AtALMT1* expression levels, as well as slight increases in *AtMATE* expression levels (Kobayashi et al., 2013). Additionally, an acidic external environment (i.e., pH approximately 4.5) leads to transcriptome-level changes in *A. thaliana* roots that resemble the transcriptomic changes induced by short-term auxin treatments. Because most Al-inducible genes are also inducible by acidic conditions (Sawaki et al., 2009), the cross-talk between auxin and low pH/Al responses may be important for the regulation of Al- and proton-tolerance genes.

### Positive Effects of ABA on Root Growth under Al Stress Conditions and the Consequences for Transcriptional Regulation

Previous studies have examined the endogenous accumulation of ABA in response to Al in buckwheat (Reyna-Llorens et al., 2015), soybean (Shen et al., 2004), and barley (Kasai et al., 1993). The application of exogenous ABA can activate the release of OAs from the roots of some plants (Ma et al., 2001; Shen et al., 2004). Hou et al. (2010) reported that Al-induced soybean root growth inhibition is alleviated in plants treated with exogenous ABA. This alleviation is suppressed by the addition of an ABA biosynthesis inhibitor (e.g., furidone). These results imply that ABA modulates Al-tolerance mechanisms, possibly through the transcriptional regulation of Al-tolerance genes. In fact, *AtALMT1* and *ALS3* expression levels are upregulated by ABA (Kobayashi et al., 2013). Additionally, the *AtALMT1* promoter differentially regulates Al and ABA responses (Kobayashi et al., 2013). Furthermore, ABA activates the release of malate from roots. These results indicate that ABA can activate *ALMT1* expression as well as malate transport activity, suggesting that Al-induced ABA accumulation induces OA transporter gene expression and activates the resulting protein (**Figure 3**). These processes may have important functions in the Al-tolerance mechanism of plants.

A comparative *A. thaliana* microarray revealed that the expression of several ABA-responsive genes (e.g., *DREB1A* and *DREB1A*-regulated genes) is induced by Al treatments (Sawaki et al., 2016). Although the consequences of the upregulated expression of *DREB1A*-regulated genes have not been studied in terms of Al tolerance, they may include increased drought tolerance that can overcome the effects of Al-induced root growth inhibition.

## Reactive Oxygen Species-Mediated Transcriptional Regulation of Al-Tolerance Genes Under Al Stress Conditions

**FIGURE 4:**
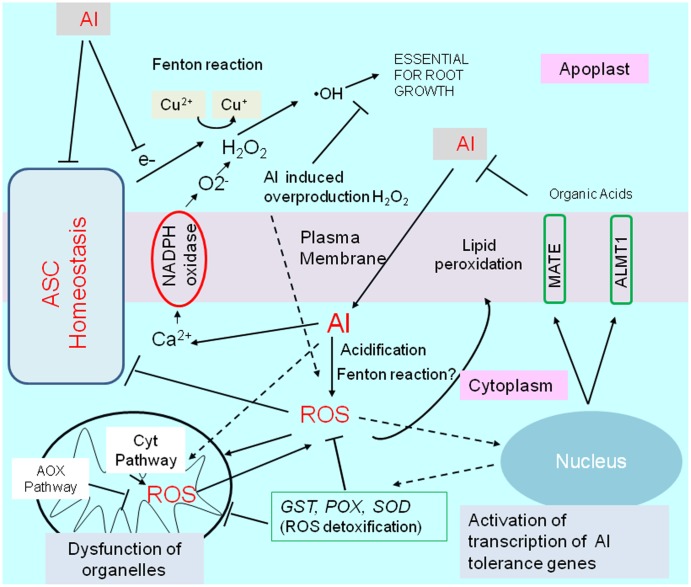
Schematic representation of aluminum-induced reactive oxygen species (ROS) stress, and activation of tolerance mechanisms by ROS. Aluminum disturbs an essential fenton reaction in the cell wall for root growth, but is induced ROS production by (1) increases cytosolic Ca^2+^ concentrations, which triggers activation of NADPH-oxidase, (2) inducing fenton reaction in cytosol, (3) cytosol acidification, and by other mechanisms. Disturbance of ascorbate (ASC) homeostasis enhance abnormal fenton reaction in apoplast, and ROS production in the cytoplasm. Aluminum (or by generated ROS) shifts mitochondrial respiration to an alternative pathway (AOX pathway) from Cyt pathway (cytochrome pathway), which is likely important to protect mitochondrial function. ROS activates transcription of several genes for ROS detoxifying enzymes such as *GLUTATHIONE–S-TRANSFERASE* (GST); *PEROXIDASE* (*POX*); *SUPEROXIDE DISMUTASE* (*SOD*), which can confer Al resistance in some plant species by overexpression. ROS, in particular, H_2_O_2_ is known as a inducer of an Al tolerance genes such as *ALUMINUM ACTIVATED MALATE TRANSPORTER1* (*ALMT1*) and *MULTIDRUG AND TOXIC COMPOUNDS EXTRUSION* (*MATE*) in Arabidopsis.

Exposure to Al stress conditions alters the cellular ROS levels in different root regions. ROS, including hydrogen peroxide (H_2_O_2_), superoxide (O_2_^-^), and hydroxyl radicals (^•^OH), adversely affect root cells (e.g., apoptosis and damages to various molecules) and influence signaling pathways for essential processes such as growth and stress adaptations (e.g., activation of Al-tolerance gene expression). In this section, we describe ROS production under Al stress conditions as well as the transcriptional regulation of plant ROS responses to mitigate ROS-induced damages (**Figure 4**).

### Reactive Oxygen Species Accumulation and Toxicity under Al Stress Conditions

The enhanced ROS production induced by Al stress conditions is mediated by several mechanisms. In the apoplast, activated plasma membrane NADPH oxidase is the primary source of ROS in Al-stressed plants. The Al transiently increases the cytoplasmic Ca^2+^ concentration (Bhuja et al., 2004), which activates the plasma membrane NADPH oxidase, leading to the production of O_2_^-^ (Sagi and Fluhr, 2001) and H_2_O_2_ in the apoplast. Under normal conditions, the Cu-mediated Fenton reaction is critical for generating ^⋅^OH and loosening the cell wall, which is essential for cell elongation, because it can cleave the sugar–sugar bond in cell wall polysaccharides. Normal Fenton reaction activity is regulated by the coupling of Cu^2+^ ↔ Cu^+^ of the cell-wall–localizing blue-copper–binding proteins and ascorbate (ASC) ↔ monodehydroascorbate (MDHA). However, Al can activate the Fenton reaction by coupling with other metals, including Cu, leading to the excessive formation of the ^•^OH radical (Mujika et al., 2011; Ruipérez et al., 2012). In contrast, a shortage of ASC in the apoplast, which may result from the excessive conversion of ASC to oxalate (Al-responsive gene; Hamel et al., 1998), leads to the accumulation of H_2_O_2_. In fact, Al treatments generate diverse ROS in the apoplast (Maltais and Houde, 2002), while the maintenance of high levels of ascorbate is characteristic of Al tolerance in tobacco and rice plants (Devi et al., 2003; Guo et al., 2005). A comparison of wheat transcriptomes revealed that Al-tolerant varieties exhibited considerably upregulated expression of genes encoding proteins belonging to such a system, including enzymes involved in ASC-homeostasis and cell-wall–loosening proteins associated with the Fenton reaction (Houde and Diallo, 2008).

Plants treated with Al usually accumulate ROS in the symplast (e.g., accumulation of H_2_O_2_ in Al-sensitive varieties; Kobayashi et al., 2005), leading to the peroxidation of lipids in the plasma membrane and the production of dysfunctional organelles (Yamamoto et al., 2002). In tobacco, Al stress is associated with swollen/dysfunctional mitochondria, fragmented vacuoles, and pre-apoptotic nuclear structures (Yamamoto et al., 2002; Panda et al., 2008), which may ultimately induce the mitochondrial pathway to initiate programmed cell death (Huang et al., 2014). The manner in which Al induces the production of excess ROS in the cytoplasm (symplast) is complex. The H_2_O_2_ accumulating in the apoplast due to NADPH oxidase or the Fenton reaction may be introduced to the cytosol (Bienert et al., 2006). Additionally, Al can quickly cross the plasma membrane (Taylor et al., 2000) and activate the Fenton reaction in the cytoplasm. These mechanisms increase the cytosolic ROS concentration. However, ROS production in the cytosol and mitochondria may also be enhanced by the acidification of the cytosol by Al (Moseyko and Feldman, 2001). This acidification disrupts the redox metabolic activities in the cytosol by inactivating -SH residues (pH < 7) and destabilizing NAD^+^. It also enhances ROS toxicity in the cytoplasm, and inhibits the production of excess ROS in mitochondria. Disrupting the redox molecules (e.g., -SH, NAD^+^) in the cytosol interferes with the regulation of NAD(P)H/NAD(P)^+^ contents in other cellular components, including mitochondria. Moreover, the generation of toxic O_2_^-^ in mitochondria is enhanced by a relatively low ATP demand or a high NADH/NAD^+^ ratio (Murphy, 2009). Under the crisis, tolerant cultivars synthesize cysteine-rich proteins to reduce ROS production (Hamel et al., 1998), while one of such proteins had been identified as an Al tolerance gene regulated the transcription by STOP1-like protein (ART1) in rice (Xia et al., 2013).

### Inducible Expression of ROS-Mediated Al-Tolerance Genes

Aluminum treatments upregulate the expression of various genes that help plants survive the effects of ROS stress/damages. A transcriptome analysis confirmed that Al induces the expression of several genes to decrease ROS production, detoxify ROS, and stimulate the recovery from ROS-induced damages (Richards et al., 1998; Kumari et al., 2008; Chowra et al., 2017). Most of these genes are responsive to diverse biotic and abiotic stresses because their expression is induced by ROS. However, some of these ROS-related genes have an active role in Al-tolerance mechanisms.

The ectopic expression of several genes confers Al tolerance to *A. thaliana* and several crops (Inostroza-Blancheteau et al., 2012). This suggests that genes encoding ROS-scavenging proteins may be useful for breeding transgenic crops that are tolerant to Al stress conditions. For example, transgenic *A. thaliana* plants overexpressing three glutathione *S*-transferase genes and two peroxidase genes from tobacco, all of which are inducible by Al, were observed to be tolerant to Al stress conditions (Ezaki et al., 2000). Additionally, the ectopic expression of wheat *WMnSOD1* confers Al tolerance to transgenic mustard plants (Basu et al., 2001). Meanwhile, Panda et al. (2013) reported that the overexpression of alternative oxidase enhances Al resistance. The shift in the regular electron transfer reaction of mitochondria (Cyt pathway) to the alternative oxidase pathway decreases ROS production under stress conditions. These results indicate that Al-inducible ROS-mediated genes help protect plants from Al-induced ROS damages.

The metabolic engineering of redox metabolic activities is another potential approach for improving Al tolerance in terms of ROS damages. As described above, the metabolism of ascorbate and tissue ascorbate levels affect ROS production in the apoplast. Yin et al. (2010) demonstrated that the overexpression of a dehydroascorbate reductase gene increases the ascorbate levels in tobacco and enhances Al tolerance. This may explain the results of the wheat transcriptome comparisons that indicated genes related to the metabolism of ascorbate were more highly expressed in Al-tolerant plants than in WT plants (Houde and Diallo, 2008). Manipulating non-enzymatic antioxidant defense molecules also improved Al tolerance, while downregulating polyamine synthesis resulted in Al sensitivity (Nezames et al., 2012). Another study concluded that the application of exogenous polyamine improved Al tolerance in saffron plants (Chen et al., 2008). This is likely because polyamines have protective roles against ROS (Alcazar et al., 2010). In contrast, the overexpression of a gene encoding an MDH exhibiting a unique kinetic property conferred Al tolerance to alfalfa plants (Tesfaye et al., 2001). This MDH converted oxaloacetate to malate, meaning its function is to convert NADH to NAD^+^. The overexpression of another gene encoding a plastid-localized MDH with a similar kinetic property also conferred Al tolerance to transgenic *A. thaliana* plants (Li et al., 2016). The transgenic lines exhibited an enhanced reducing capacity for 2,3,5-triphenyl tetrazolium chloride, indicating that increased respiration may improve Al tolerance. Finally, it is important to note that Al-induced ROS production can also activate the transcription of several Al-tolerance genes. For example, H_2_O_2_ induces the transcription of *AtALMT1* and *AtMATE* in *A. thaliana* (Kobayashi et al., 2013). Furthermore, *SbMATE* (i.e., citrate transporter in sorghum) expression is upregulated by the accumulation of ROS, which serves as an indicator of Al exposure (Sivaguru et al., 2013).

## Concluding Remarks and Future Interests

Recent studies clarified the complex transcriptional regulation of Al-tolerance genes (**Table 1**). The expression of major Al-tolerance genes, such as *ALMT1* and *MATE*, is regulated by STOP1-type zinc-finger proteins. The STOP1-regulated system likely also affects proton tolerance, plant immunity, and root development under P-starvation conditions. The transcription of major Al-tolerance genes, including *ALMT*, is also regulated by phytohormones and ROS. These signal inducers regulate Al tolerance via the transcriptional regulation of diverse genes. However, recent studies also have shown that abnormal accumulation of phytohormones (e.g., IAA) is involved in the Al-induced suppression of root growth, which is concomitant with transcriptional regulation of various genes. These findings clearly indicate there is crosstalk between the transcription of Al-tolerance genes and various stress response mechanisms. Future studies should characterize the molecular mechanisms underlying this crosstalk. Additionally, the mechanisms regulating specific responses to Al will need to be elucidated. Combination of genome-wide approaches such as genome-wide association study and expression level polymorphism analysis, and its integration with genome-wide functional genomics may be useful to elucidate true nature of complex transcriptomic regulation of Al tolerance.

**Table 1:** Summary of transcriptionally regulated Al tolerance genes, and their regulatory genes mediating Al, phytohormone and reactive oxygen species signaling.

Gene name	Function	Plant species
**Transcription factor**		
*STOP1-like proteins*	Regulates expression of Al tolerance genes	Arabidopsis (*AtSTOP1*, Iuchi et al., 2007), Tobacco (*NtSTOP1*, Ohyama et al., 2013), Rice bean (*VuSTOP1*; Fan et al., 2015), Rice (*ART1* Yamaji et al., 2009)
*Others*	Repressor of *AtALMT1*	*AtWRKY46* (Ding et al., 2013)
	Activator of *AtALMT1*	*AtCAMTA2* (Tokizawa et al., 2015)
**Organic acid transporters**		
*ALMT1*	Al activated malate transporter	Arabidopsis (*AtALMT1*^∗^, Hoekenga et al., 2006), *Holcus lanatus* (*HlALMT1*, Chen et al., 2013)
*MATE*	Al activated citrate transporter	Sorghum (*SbMATE*, Magalhaes et al., 2007), Arabidopsis (*AtMATE1*^∗^, Liu et al., 2009), Rice (*OsFRDL4*^∗^, Yamaji et al., 2009), Rice bean(*VuMATE1*^∗^, Fan et al., 2015)
**Other transporters**		
*ALS3*	UDP-glucose transporter (?)	Arabidopsis (*AtALS3*^∗^, Larsen et al., 2005), Rice (*OsSTAR2*^∗^, Yamaji et al., 2009), Tobacco (*NtALS3*^∗^, Ohyama et al., 2013)
*MGT*	Magnesium transporter	Rice (*OsMGT1*^∗^, Chen et al., 2012)
**Other Al responsive genes**		
*OsCDT3*	Cys-rich peptide at PM (Alter Al binding to the PM)	Rice (*OsCDT3*^∗^, Xia et al., 2013)
**Al-mediated phytohormone responsive genes**		
*PIN*	Auxin polar transporters	Arabidopsis (At*PIN2*, Shen et al., 2008), Rice (*OsPIN2*, Wu et al., 2014)
*TAA1/YUCCA*	Auxin biosynthesis	Arabidopsis (*TAA1*/*YUCCA*, Liu et al., 2016)
**Al inducible ROS detoxifying enzymes**		
*GST*	Glutathione S-Transferase	Arabidopsis (*AtGST*, Ezaki et al., 2000)
*POX*	Peroxidase	Tobacco (*NtPOX*, Ezaki et al., 2000)
*MnSOD1*	MnSOD	Wheat (*WMnSOD*, Basu et al., 2001)


*Asterisks indicate transcriptional regulation by STOP1-like proteins.*

## Author Contributions

AD wrote the article, revised the text based on feedback from the co-authors, and prepared the illustrations. AS and MT helped prepare the figures. SP provided editorial suggestions regarding the article. HK and YK conceptualized the overall structure of the review article and critically edited it. All authors have read and approved the final draft.

## Conflict of Interest Statement

The authors declare that the research was conducted in the absence of any commercial or financial relationships that could be construed as a potential conflict of interest.
